# Subcutaneous Emphysema and Pneumomediastinum Following Non-invasive Ventilation in a Patient With Severe COVID-19 Disease

**DOI:** 10.7759/cureus.16051

**Published:** 2021-06-30

**Authors:** Ravichandra Tata, Thrilok Chander Bingi, Akhilesh Kumar Maurya, Hemanth Kalakuntla, Saketh Gangishetti

**Affiliations:** 1 General Medicine, Gandhi Hospital, Hyderabad, IND

**Keywords:** subcutaneous emphysema, pneumomediastinum, non-invasive ventilation, covid-19, sars-cov-2

## Abstract

Subcutaneous emphysema (SE) and pneumomediastinum are rare complications of severe acute respiratory syndrome coronavirus 2 (SARS-CoV-2). While SE is often non-fatal and usually self-remitting, pneumomediastinum can be fatal with high mortality rates depending on the underlying etiology. Here, we present the case of a 39-year-old otherwise healthy male who tested positive for SARS-CoV-2. The patient was treated with non-invasive mechanical ventilation (NIMV) and developed severe SE and pneumomediastinum which resulted in a fatal outcome. Although the exact pathogenesis could not be determined, the extensive lung injury caused by SARS-CoV-2 pneumonia along with possible barotrauma secondary to NIMV could have been the culprits in this case.

Early detection through careful observation of these potentially fatal complications in patients with severe coronavirus disease 2019 is crucial. Further studies determining the potential risk factors and incidence of SE and pneumomediastinum, especially in patients receiving invasive mechanical ventilation or NIMV, are needed.

## Introduction

Since its outbreak in December 2019, coronavirus disease 2019 (COVID-19) has become a pandemic spreading across 215 countries and territories. Caused by the severe acute respiratory syndrome coronavirus 2 (SARS-CoV-2) virus, it has claimed over 12 million lives globally [1].

Diffuse alveolar injury to type I and II pneumocytes in severe COVID-19 pneumonia is a characteristic finding, which may make the alveoli more susceptible to rupture, especially as patients often present with pronounced cough [2]. Although rare, there have been sporadic reports of the occurrence of subcutaneous emphysema (SE) in COVID-19 patients, often associated with pneumothorax and/or pneumomediastinum [3,4].

Here, we present the case of a patient treated with non-invasive mechanical ventilation (NIMV) who developed pneumomediastinum and SE involving multiple fascial planes.

## Case presentation

A 39-year-old male, non-smoker, with no known comorbidities, and history of high-grade fever, persistent dry cough, and progressive dyspnea for three days and a positive test for SARS-CoV-2 was admitted to our intensive care unit (ICU) due to hypoxic respiratory failure. On admission, the patient’s temperature was 38.2°C, heart rate was 120 beats per minute, blood pressure was 130/77 mmHg, respiratory rate was 42 breaths per minute, and oxygen saturation (SpO_2_) was 70% on room air. An initial chest X-ray revealed ground-glass opacifications of both lung fields.

The patient was immediately started on NIMV with the following settings: FiO_2_ of 70%, positive end-expiratory pressure (PEEP) of 8 cm H_2_O, and pressure support (PS) of 10 cm H_2_O. His SpO_2_ gradually improved to 95%. An arterial blood gas measurement indicated severe acute respiratory distress syndrome (ARDS) with a PaO_2_/FiO_2_ ratio of 98.5. Intravenous remdesivir, 200 mg loading dose followed by 100 mg once daily, antibiotics, and a short course of low-dose dexamethasone was started.

**Figure 1:**
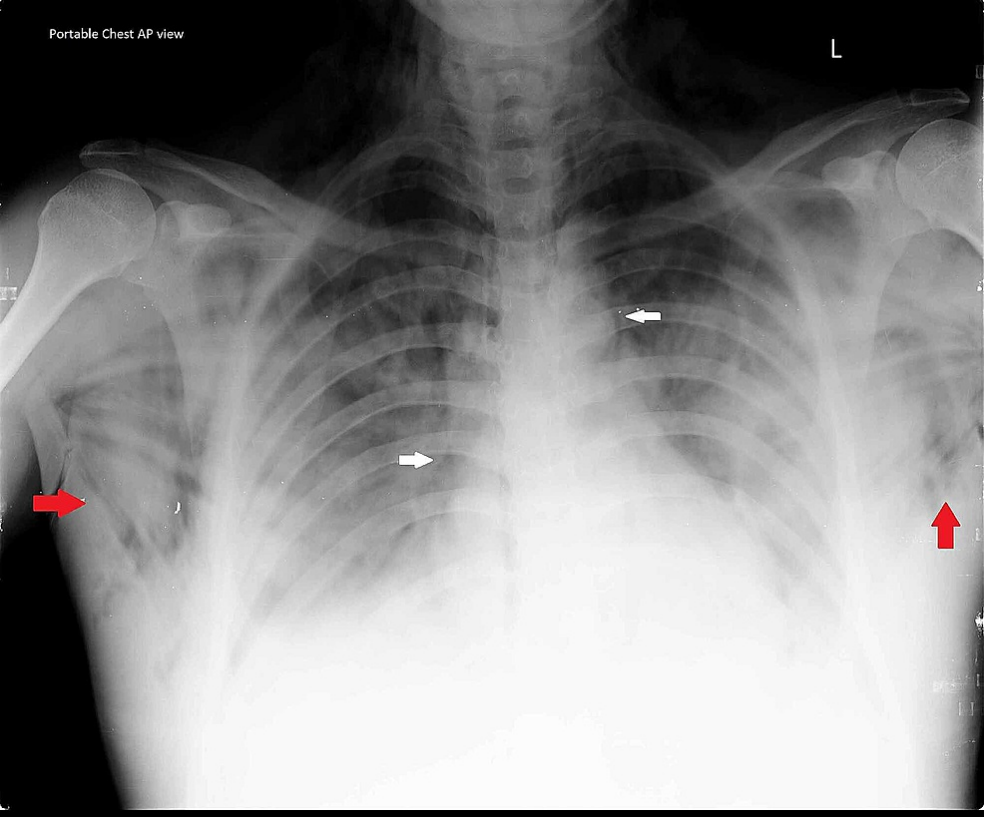
Chest X-ray shows bilateral ginkgo leaf sign indicating SE (red arrows) and air in the mediastinal pleura indicating pneumomediastinum (white arrows). SE: subcutaneous emphysema

On the second day of admission, the patient’s work of breathing increased, with failure in maintaining SpO_2_. Hence, PEEP was increased to 12 cm H_2_O, PS to 14 cm H_2_O, and FiO_2_ was set to 80%. Shortly thereafter, he developed chest pain with increasing difficulty in breathing and a steady fall in SpO_2_. In addition, crepitus was noted over the neck and upper thoracic region along with a fall in SpO_2_. Chest X-ray taken revealed a severe SE of the chest wall with characteristic bilateral “ginkgo leaf” sign (Figure 1), brought about by air in the subcutaneous tissue and around the pectoralis major creating radiolucent striations of the individual muscle fibers [5]. Pneumomediastinum was also noted with absent pneumothorax.

Infraclavicular blowholes about 3 cm wide were made bilaterally to relieve the SE. This improved the patient’s symptoms and his oxygen saturation. The blowholes were dressed using sterile gauze, taped on three sides, leaving the fourth side open to create a one-way valve to allow continuous expulsion of air. PEEP was readjusted to 10 cm H_2_O.

Recurrence of SE was noted on the third day of admission, now extending to the patient’s face and his entire upper body and arms. His SpO_2_ dropped to 52% with PaO_2_ of 52.5 mmHg. Surgical consultation was sought and bilateral intercostal subcutaneous drains at the fifth intercostal space were placed.

There was no improvement in his condition over the next couple of days. Upon discussion of further management options, the family refused consent for the use of any extraordinary measures including invasive mechanical ventilation. He eventually passed away on day six of hospitalization.

## Discussion

SE is the collection of air in the subcutaneous layer of the skin just beneath the dermis and/or in the soft tissues, most commonly occurring in the chest wall or neck region. On the other hand, pneumomediastinum is the presence of air in the mediastinum. Etiologies include spontaneous onset, trauma to the thoracic cavity, secondary to ARDS, mechanical ventilation, tracheobronchial injury, cardiothoracic interventions, esophageal rupture, asthma, parturition, chronic lung diseases, Valsalva maneuvres, infections, malignancy, complications during surgical procedures, and iatrogenic injuries such as ventilator-associated barotrauma [6-8]. Although usually mild, SE can evolve into a life-threatening condition [6], especially when occurring along with pneumomediastinum or pneumothorax [9].

SE and pneumomediastinum are rare complications of COVID-19 infection. Although the exact incidence is not yet known, few cases of SE and spontaneous pneumomediastinum associated with or without positive pressure ventilation in patients with COVID-19 have been reported [10-12]. One such report was of a 78-year-old male, a heavy smoker, who died after developing pneumomediastinum secondary to NIMV [13]. NIMV has become a popular choice in treating ARDS due to its advantages over invasive mechanical ventilation. Although barotrauma is a known complication of pressure support ventilation, its risk in NIMV is very low. Barotrauma can also occur in the presence of underlying lung pathology such as acute lung injury secondary to pneumonia [14].

The exact pathogenesis underlying SE and pneumomediastinum in COVID-19 is yet to be determined. However, in the current case, extensive alveolar injury of type I and II pneumocytes due to COVID-19 infection, increased intrathoracic pressure generated by the NIMV, and persistent cough of the patient may have caused a rupture of the alveoli, leading to the occurrence of SE and pneumomediastinum [11,13].

## Conclusions

Although rare complications of COVID-19, SE and pneumomediastinum may occur spontaneously or secondary to invasive or non-invasive mechanical ventilation, and can be predictors of poor prognosis. Further studies are needed to investigate the underlying incidence, risk factors, and pathogenesis of these rare yet potentially fatal complications of COVID-19 pneumonia. In the face of the current pandemic, it is recommended that physicians lookout for such potentially fatal complications in patients admitted to ICUs, especially in those requiring NIMV or invasive ventilation.
